# Order Patterns Networks (ORPAN)—a method to estimate time-evolving functional connectivity from multivariate time series

**DOI:** 10.3389/fncom.2012.00091

**Published:** 2012-11-07

**Authors:** Stefan Schinkel, Gorka Zamora-López, Olaf Dimigen, Werner Sommer, Jürgen Kurths

**Affiliations:** ^1^Department of Physics, Humboldt-Universität zu BerlinBerlin, Germany; ^2^Department of Psychology, Humboldt-Universität zu BerlinBerlin, Germany; ^3^Bernstein Center for Computational NeuroscienceBerlin, Germany; ^4^Potsdam Institute for Climate Impact ResearchPotsdam, Germany; ^5^Institute for Complex Systems and Mathematical Biology, University of AberdeenAberdeen, UK

**Keywords:** functional networks, network reconstruction, order patterns, time series analysis, EEG, ERP, semantic priming

## Abstract

Complex networks provide an excellent framework for studying the function of the human brain activity. Yet estimating functional networks from measured signals is not trivial, especially if the data is non-stationary and noisy as it is often the case with physiological recordings. In this article we propose a method that uses the local rank structure of the data to define functional links in terms of identical rank structures. The method yields temporal sequences of networks which permits to trace the evolution of the functional connectivity during the time course of the observation. We demonstrate the potentials of this approach with model data as well as with experimental data from an electrophysiological study on language processing.

## 1. Introduction

**Figure 1:**
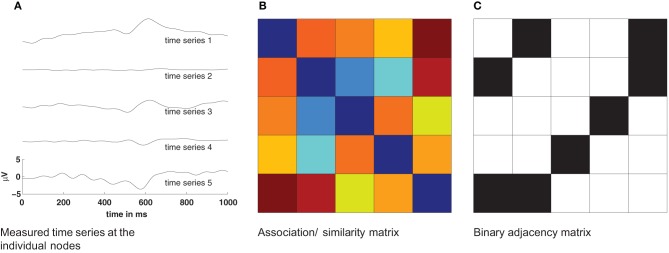
**Basic procedure for estimation of networks from time series**. The pairwise similarity of all measured time series **(A)** is computed and provides an association/similarity matrix **(B)**. By applying a threshold to such an association matrix a binary adjacency matrix *A*_*ij*_ is estimated **(C)**. This adjacency matrix is then used to compute statistical properties of the network.

Complex networks provide an excellent framework for studying the function of the human brain on a variety of scales, from the interaction of single neurons to the activation of large cortical areas. Plenty of studies have recently shown the merit of a graph-theoretical approach to better understand brain functions (Zhou et al., 2006; Stam and Reijneveld, 2007; Bullmore and Sporns, 2009; He and Evans, 2010; Zamora-López et al., 2011). However, estimating functional networks from measured signals of brain activity is far from trivial, especially if the data is non-stationary and noisy. In the process of deriving functional networks from a set of time series some critical decisions have to be made (Figure 1). First, one has to decide which dynamical measure to apply for estimating the functional connectivity. This choice defines the nature of the dynamic interactions considered as a functional connection. Usually this step gives a real-valued all-to-all functional connectivity matrix between all nodes (Figure 1B). However, measures to characterize the network properties often require a binary connectivity matrix. Therefore, as a second step, a threshold is usually applied to convert the real-valued similarity matrix into a binary matrix representing the functional graph (Figure 1C). In the absence of standard criteria, this step is prone to arbitrary choices and to the convenience of the users, what influences the results (van Wijk et al., 2010).

There are several methods to estimate the similarities and/or the causality between two or more time series, e.g., linear correlation, Granger Causality (Granger, 1969), partial directed coherence (Baccala and Sameshima, 2001), and partial phase synchronization (Schelter et al., 2006; Nawrath et al., 2010). Practical applications of most of the available methods require rather long and, at best, stationary data. This is rarely the case in (electro-)physiological studies, with the prominent exception of resting-state data. The aim of most medical and psychological studies is to investigate the responses of the brain to sensory stimuli, often triggering further cognitive processing. Consequently, the data obtained in such studies is characterized by short and transient non-stationarities for which most of available methods are not suitable.

As an effort to cover this gap, we introduce a method to estimate functional connectivity between time series that is suitable for short and non-stationary datasets and is computationally efficient. Moreover, it permits to study the temporal evolution of the functional connectivity along the course of the measurements. The method employs the concept of order patterns, which provides a symbolic representation of a real-valued time series in terms of its local rank structure. Order patterns have been shown to be suitable for short and non-stationary data before (Bandt and Pompe, 2002; Schinkel et al., 2007; Staniek and Lehnertz, 2008; Hempel et al., 2011; Martini et al., 2011). In the present formulation of the method we replace the notion of similarity (Figure 1B) by that of identity such that the method directly returns binary connectivity matrices (Figure 1C). This avoids the need to choose a threshold to obtain the binary functional links. Relaxation of this constraint will permit to obtain similarity values if the user so desires.

The remainder of the paper is organized as follows. First, we review the concepts of symbolic dynamics and order patterns in the analysis of single time series. Second, we explain how to obtain the time-evolving functional networks from multivariate time series. And third, we show practical examples of the method. Its accuracy is compared to that of correlation using simulated data of coupled Lorenz oscillators. We illustrate the convenience of our method for neurophysiological studies by applying it to characterize the event-related brain potentials from a semantic priming experiment, which is a well-known experimental paradigm in the psychological literature.

## 2. Order patterns in time series

The analysis of symbolic time series has repeatedly been proven suitable for the investigation of physiological data (Kurths et al., 1995; Lind and Marcus, 1995; beim Graben et al., 2000; Daw et al., 2003). The general idea is to encode a given time series of real values, e.g., a measured signal, into a time series of symbols, where the symbols stand for a more abstract and/or coarse-grained representation of the data. Conceptually, such a transformation represents a partition of the phase space of the dynamical system into a small number of regions. When the time series changes from one symbol to another, it corresponds to a transition of the system from one state to another. The resulting sequence of symbols is easier to analyze because the discretization will yield only a reduced set of states.

In the present case we use a particular type of symbolic representation, which captures the local order structure of a trajectory by comparing whether the values of consecutive data points increase or decrease. Given a time series {*u*(*t*)} where *t* = 1, 2, …, *T*, the simplest order patterns we can define are of dimension *d* = 2. The dimension denotes the number of data points participating in the order pattern. Consider two instances of the series, *u*(*t*) and *u*(*t* + τ), separated by a delay τ. If the second value is higher than the first one, this pattern is encoded as 0, and if the second value is lower, the pattern is encoded as 1:

(1) $$\pi (t)=\{\begin{array}{l} 0:u(t)<u(t+\tau )\\ 1:u(t)>u(t+\tau ) \end{array}$$

Tied ranks, *u*(*t*) = *u*(*t* + τ), are rare in real-valued time series and can be neglected. Encoding every point of the signal {*u*(*t*)}, a time series of symbols {π(*t*)} of length (*T* − (*d* − 1)τ) is obtained.

**Figure 2:**
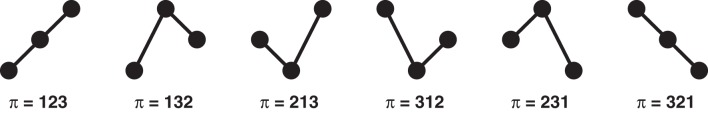
**Sample order pattern for *d* = 3**. If ties are neglected the number of possible patterns is *d*!.

For dimension *d* = 3, three instances are considered for comparison, namely, *u*(*t*), *u*(*t* + τ), and *u*(*t* + 2τ). In this case, and ignoring again tied ranks, there are six different possible patterns as shown in Figure 2 which are encoded according to the permutation of the rank indices. Consider the relation *u*(*t*) < *u*(*t* + 2τ) < *u*(*t* + τ): it is described by the order pattern π = 132 since *u*(*t* + τ) is the largest, *u*(*t* + 2τ) the second largest and *u*(*t*) the smallest of the three values. In general, for dimension *d* there are *d*! order patterns.

The only two parameters required are the dimension *d* and the delay τ. Established algorithms for the estimation of both are available. The dimension can be estimated using the method of *false nearest neighbors* (Kennel et al., 1992) and the delay is commonly estimated using the auto-correlation or the mutual information function (Roulston, 1999).

The resulting time series of order patterns, {π(*t*)}, are rather robust against observational noise, nonlinear amplitude distortions and low-frequency trends. The reason is that order patterns capture the dynamical behavior of a time series {*u*(*t*)} by describing its shape, regardless of the precise value of the amplitude. Therefore, they are almost unaffected by small fluctuations of the amplitude, e.g., caused by additive noise, and they are robust with respect to slow variations of the original signal (Groth, 2005).

In the following, we introduce the steps to obtain functional networks from multivariate time series using order patterns.

## 3. Networks based on series of order patterns

**Figure 3:**
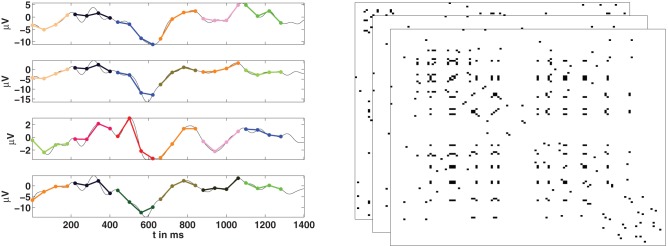
**Estimation of functional networks from multivariate time series**. **Left:** For the time series of each channel, its corresponding series of order patterns is computed. Here some example order patterns of dimension four are shown as encoded by colors. If at a given time *t*, two nodes are encoded by the same pattern, then they are considered to be functionally connected. **Right:** Repeating this for all nodes, at all time-points, we obtain a temporal sequence of adjacency matrices.

Given a set of *N* time series we can now use the concept of order patterns to obtain a temporal sequence of networks, as is illustrated in Figure 3. If {*u*_*i*_(*t*)} is the signal of the channel (node) *i*, we first need to compute the corresponding series of order patterns {π_*i*_(*t*)} for all the channels. Then, the sequence of adjacency matrices {*A*(*t*)} is constructed using the symbolic sequences {π_*i*_(*t*)}. At every time point two channels *i* and *j* are considered as functionally connected if they are encoded by the same symbol:

(2) $$A_{ij}(t)=\{\begin{array}{l} 1:\;\pi_{i}(t)=\pi_{j}(t)\\ 0:\;\pi_{i}(t)\neq \pi_{j}(t) \end{array}$$

The result is a sequences of *N* × *N* binary (unweighted) adjacency matrices of length *T* − τ, the same length as the series of symbols. From this sequence one can calculate network properties over the time course of the observation. The identity criterion in Equation (2) implies that all nodes encoded by the same symbol are connected together and disconnected from all other nodes, leading to networks which are segregated into different components.

### 3.1. Estimation of the parameters

The only two parameters required to compute the order patterns can be obtained following established methods. The dimension *d*, is estimated by the false nearest neighbours (FNN) method (Kennel et al., 1992). The FNN algorithm checks for the neighborhoods of points which are embedded in projection manifolds. As the dimension of the projection is increased, apparent neighbors are separated until only “true” neighbors remain (Kantz and Schreiber, 1997). In general, it is recommended to use a higher dimension than estimated because over embedding helps coping with non-stationarity (Hegger et al., 2000) and from now on we will consider a modified dimension $\hat{d}$ = 2*d* + 2. We will illustrate this issue with an example in the following section.

The delay τ is estimated using the first minimum in mutual information function (Cover and Thomas, 2006). Therefore, the (auto-)mutual information function between a time series {*u*(*t*)} and its time-lagged counterpart {*u*(*t* + τ′)} is computed for a range of τ′s:

(3) MI(u(t), u(t+τ′))

The delay τ′ giving rise to the first minimum of this function is taken as the embedding delay. A relevant issue arises from the fact that in order to compute the mutual information the data has to be binned into histograms. The delay estimates depend on the number of bins used. If too few bins are used, the delay is overestimated. The estimates of the delay converge after a sufficiently high number of bins. We will show this dependency in the example of electroencephalographic (EEG) data. In that case 100 bins are sufficient.

When multiple observations or realizations are available as it happens in multivariate time series, the parameters *d* and τ have to be computed for all the time series. There is a certain trade-off between accuracy and generality. According to our experience with EEG data, the variation in the estimates of the dimension and of the time delays across channels are reasonably small. Therefore, we choose the mode of the dimension estimates for the whole system and for the delay, we use the mean of the individual estimates. In combination with over embedding this appears to be a suitable compromise.

Since the order patterns span a certain time range, Δ*t* = ($\hat{d}$ − 1) τ, the timescale of the measurement has to be re-aligned. This is done in such a way that t^*^ is *t* + Δ*t*/2. This effectively means that the time point t^*^_0_ is exactly in the middle of the window spanned by the pattern π(*t*_0_) as is commonly done in a windowed analysis.

In the following sections we show the application of the procedure described here and revisit some of the technical issues discussed.

## 4. Application of the ORPAN method

In this section we show the application of the orpan method to estimate functional connectivity in two examples. First, we apply the procedure to simulated data from a simple model of two Lorenz attractors. The simplicity of the model allows us to evaluate the accuracy of the method to detect functional connections. We also investigate the benefits of over embedding for this purpose. Second, we apply the method to EEG data from a semantic priming experiment. We discuss the influence of data binning for the estimation of the embedding delay and we study the temporal evolution of the network properties.

### 4.1. Coupled lorenz attractors

A system of two coupled Lorenz attractors *x* and *y* is defined by the following equations (Lorenz, 1963):

(4) $$\begin{array}{l} {\dot{x}}_{1}=10[(x_{2}-x_{1})]+g[y_{1}-x_{1}]\\ {\dot{x}}_{2}=x_{1}[28-x_{3}]-x_{2}\\ {\dot{x}}_{3}=x_{1}x_{2}-8/3\;x_{3}\\ {\dot{y}}_{1}=10[y_{2}-y_{1}]+g[x_{1}-y_{4}]\\ {\dot{y}}_{2}=y_{1}[28-y_{3}]-y_{2}\\ {\dot{y}}_{3}=y_{1}y_{2}-8/3y_{3} \end{array}$$

were *g* is the strength of the coupling. The parameters are chosen such that both attractors are in the chaotic regime. The initial conditions were chosen such that *x* = (−1, 3, 4) and *y* = (−8, 8, 27) with random perturbation in the range [−0.5, 0.5]. The equations of motion were solved using the *ode45* algorithm available in MATLAB (2010, The MathWorks, Natick, MA). The *ode45* solver implements a solver of the Runge–Kutta family and calculates the fourth- and fifth-order accurate solutions using the Dormand–Prince method (Dormand and Prince, 1980). The equations were integrated with a step size of 0.001 and later down-sampled by a factor 1/5.

**Figure 4:**
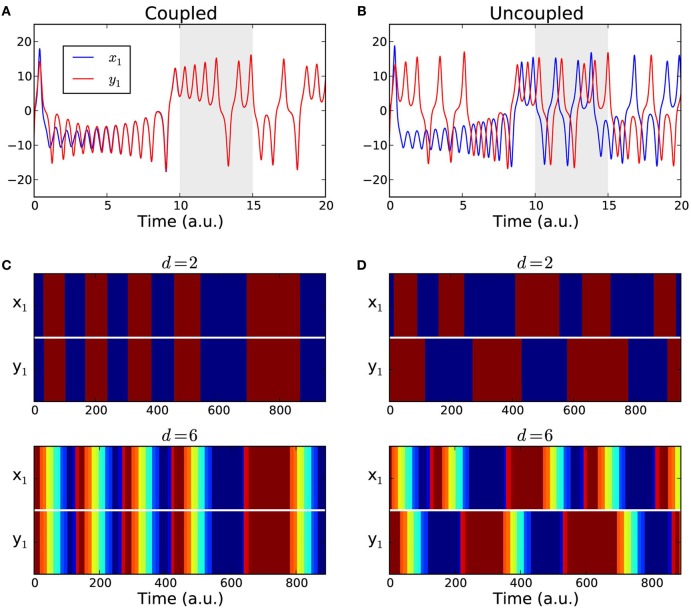
**Coupled Lorenz attractors**. **(A,B)** Sample time series of the first components *x*_1_(*t*) and *y*_1_(*t*) of two Lorenz systems. The data span used for the analysis (gray area) starts after a transient of 10,000 data points to ensure that the attractors are either fully synchronized (coupled case) or desynchronized (uncoupled case). **(C,D)** Color-coded order patterns of the time series above in the studied interval for the coupled case **(C)** and for the uncoupled case **(D)**. The sequences in the upper panels were computed using order patterns of dimension *d* = 2, and using an over embedded dimension $\hat{d}$ = 2*d* + 2 = 6 for the lower panels.

To compute the functional connectivity between the two oscillators we considered the evolution of their first components *x*_1_ and *y*_1_. We considered time series of length *T* = 1000 data points, after discarding the first 10,000 data points of the transient, Figures 4A,B. The coupling strength was set either to *g* = 0 for the uncoupled case or to *g* = 5 which ensures complete synchronization.

We converted the simulated time series into sequences of symbols {π_x_1__(*t*)} and {π_y_1__(*t*)} using the estimated parameters *d* = 2 and τ = 30. The resulting symbolic sequences are shown in Figures 4C,D with color-coded symbols. For all the *T* = 1000 time points, the presence or the absence of a functional link between the two attractors was determined by comparing their corresponding symbolic sequences as in Equation (2). The fraction of links in the interval was counted. In the coupled case we expect that the two sequences always match yielding a link detection rate of 1.0, because after an initial transient period the two attractors become fully synchronized. In the uncoupled case we expect the fraction of functional links to approach zero. As a reference, we also calculated the linear correlations between {*x*_1_(*t*)} and {*y*_1_(*t*)} in the same time intervals and in a windowed fashion, with a window of size that matched the size of the order patterns, *w* = (*d* − 1)τ. We averaged the correlation over all windows to obtain an overall detection rate. The process was repeated for 1000 realizations. The comparative results of the link detection rates are summarized in Table 1. Finally, to investigate the impact of over embedding, we repeated the numerical experiment but using $\hat{d}$ = 2*d* + 2 = 6 to compute the sequences of symbols, which are shown in Figures 4C,D (lower panels) with color-coded symbols.

**Table 1:** **Summary of the detection rate (%) of functional links between two Lorenz attractors by order patterns and by linear correlations in the coupled and in the uncoupled cases**.

**Case**	**Dimension**	**<Detection rate>**	
		**ORPAN**	**Correlation**
Coupled	*d* = 2	0.99	0.99
	$\hat{d}$ = 6	0.95	0.99
Uncoupled	*d* = 2	0.50^*^	0.86^*^
	$\hat{d}$ = 6	0.01^*^	0.55^*^

*Rates computed out of 1000 realizations. Asterisks denote the false positives in the uncoupled case. Whereas for the coupled case both correlation and order patterns detect synchronization, for the uncoupled case correlation returns a high rate of false positives. Over embedding ($\hat{d}$ = 6) significantly reduces false positives in the uncoupled case*.

We found that for the coupled case both order patterns and correlation performed quite well. In this case, over embedding had hardly any effect on either method. For the uncoupled case, however, the results differed strongly. Using only the estimated dimension (*d* = 2)^1^ the order pattern yielded a detection rate of 0.5 and the average correlation was 0.85—which in this case are *false positives*. For the over embedded case ($\hat{d}$ = 6), the detection rate for order patterns decreased significantly and became negligible (0.01), whereas correlations still yielded around 55% false positives (cf. Table 1).

Summarizing, for this prototypical example we can conclude that while correlation is suitable to detect synchronized behavior, it is clearly too error-prone for the unsynchronized case. This is reflected in the high rate of false positives in the uncoupled case. The order patterns on the other hand performed well in the coupled case and, when using over embedding, also in the uncoupled case. This shows the value of over embedding, not only to cope with non-stationarity but also to reduce the number of false positives.

### 4.2. Application to electrophysiological data

#### 4.2.1. Experimental setup and data collection

Ten adult subjects (1 male, 9 female) aged 19–38 (mean 23.6; SD 5.4 years) participated in a semantic priming experiment. All were right-handed [mean handedness index: +98 (Oldfield, 1971)] and native speakers of German. They gave written informed consent to the experiment, and received either payment or course credits. The subjects were presented with a written noun as a prime word that was either a synonym of the following target word (the primed condition), or an unrelated noun (the unprimed condition). The stimulus material was taken from (Hohlfeld et al., 2004). In total each subject read 240 items, 120 in each condition. Subjects had to indicate by a button press with either the right or the left hand, whether the target word was synonymous with the prime word or not. The response hand assigned to synonyms and non-synonyms was changed midway during the experiment. We previously used the same dataset to test other methods and to report differences in functional connectivity depending on the experimental condition (Schinkel et al., 2011). Our previous method could not estimate time-evolving connectivity as is the case for the method we now introduce.

The high degree of semantic relatedness of synonymous words as compared to the unrelated words strongly modulates the N400 component in the event-related potential (ERP). The N400 component is widely considered to reflect the retrieval of semantic word information from long term memory (Kutas and Federmeier, 2000) and its integration into the semantic context provided by the prime word. If this semantic retrieval and integration is easy, as for synonymous words, the N400 amplitude is small, whereas it is larger (up to 5 μV) when there is no such context as in the case of unrelated prime words.

**Figure 5:**
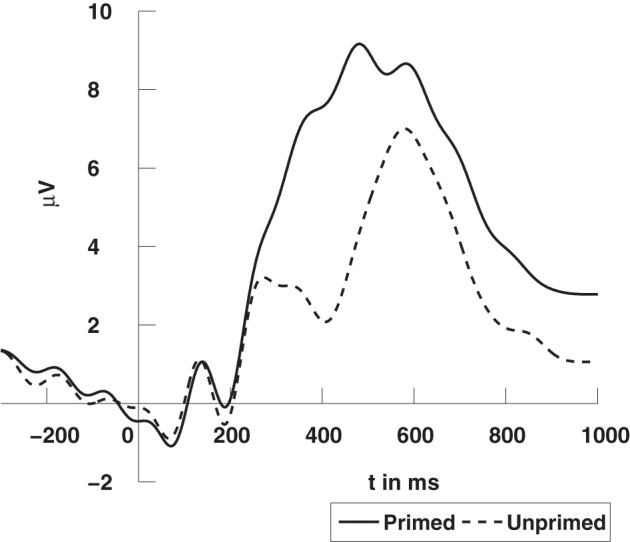
**Grand average ERPs time-locked to stimulus onset at a centro-parietal electrode (CPz)**. The N400 refers to the deflection elicited by unprimed items (dashed line) in comparison to primed items (solid line). For visualization the data was low-pass filtered at 10 Hz. The data was previously published in Schinkel et al. (2011).

The EEG was recorded from 126 Ag/AgCl electrodes (impedances ≤5 kΩ) at a sampling rate of 1000 Hz using a BrainAmp DC amplifier (Brain Products GmbH, Munich, Germany). All electrodes were initially referenced to an electrode on the left mastoid (A1) and converted to average reference off-line. The EEG data was bandpass filtered from 0.1 to 30 Hz. Trials with artifacts or an incorrect response were excluded from the analysis. One subject had to be discarded due to excessive artifacts. In a conventional analysis of ERP, we considered the average signal over trials and experimental conditions for each channel, so for every channel in every subject we obtained two average signals, one for the primed condition, and another for the unprimed condition, see example in Figure 5.

#### 4.2.2. ORPAN estimation of the embedding delay

**Figure 6:**
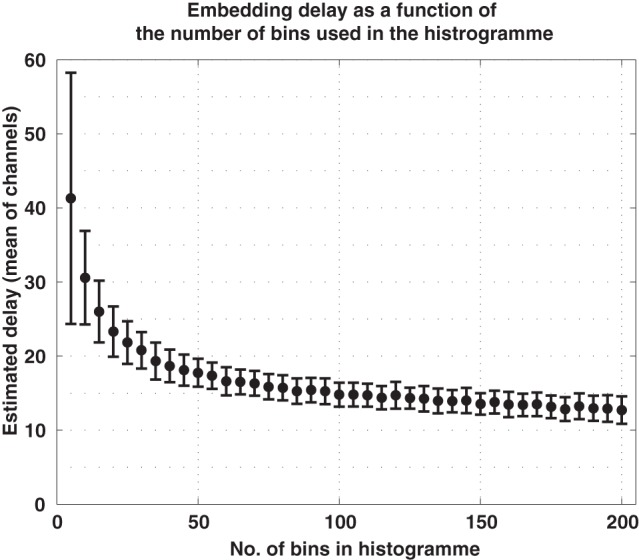
**Embedding delay of the EEG signals as a function of the number of bins used in the histograms to compute mutual information**. Too few bins lead to excessively large estimations of the delay. For the current data, the estimate converged for approximately 100 bins.

To translate the average signals of the ERP into sequences of order patterns, we first estimated the parameters *d* and τ for every channel in every subject. The dimension was estimated to be *d* = 3 (all estimates were either 2 or 3) and we used $\hat{d}$ = 8, as indicated above. We also noted that the estimation of the embedding delay τ may depend on the number of bins used for the histograms when computing the mutual information function, Equation (3). To clarify this matter we estimated τ from the EEG data using an increasing number of bins. We found out that, for a sufficient number of bins, the estimation of τ converges as shown in Figure 6. Consequently we chose to compute mutual information using 100 bins, what results in an average delay of τ = 15 ms. These parameters, $\hat{d}$ = 8 and τ = 15 ms, were then used for all subjects and for all channels.

From the series of order patterns we then computed the functional networks following Equation (2). For each subject we obtain two series of networks, one for the ERP data of the primed condition and another for the ERP data of the unprimed condition.

#### 4.2.3. Analysis of the functional networks

Now, graph measures can be applied to the sequences of functional networks to study their temporal evolution. We apply a few basic graph measures for illustration (Stam and Reijneveld, 2007; Steuer and Zamora-López, 2008). The density of links, ρ, is defined as the fraction of links in a network to the total number of possible links. If self-connections of the nodes are avoided and *L*(*t*) is the number of links in the functional network at time *t*, the density of the network is:

(5) $$\rho (t)=\frac{L(t)}{N(N-1)},$$

where *N* was the number of nodes or channels. The clustering coefficient, *C*, characterizes the probability that the neighbors of one node, are also connected with each other. It is easily understood in social terms: two persons are more likely to know each other when they have a common friend. The clustering of one node is computed as the density of links among its neighbors. The degree of a node *i* is defined as the number of neighbors it has, *k*_*i*_ = ∑^*N*^_*j* = 1_ *A*_*ij*_. Let *E*_*i*_ be the number of links between the *k*_*i*_ neighbors of node *i*. Hence, the clustering coefficient of node *i* at time *t* is:

(6) $$C_{i}(t)=\frac{E_{i}(t)}{k_{i}(t)\left(k_{i}(t)-1\right)}.$$

The clustering coefficient of the network is then the average of the individual clusterings, *C*(*t*) = 〈*C*_*i*_(*t*)〉, and it strongly depends on the density of links. Therefore, we will show the normalized clustering, *C*′(*t*) = *C*(*t*)/ρ(*t*). Indeed, the clustering of a random network is equal to its density of links, hence, *C*′(*t*) represents the deviation from the expected clustering by random chance.

The distance *d*_*ij*_ between two nodes in a network is the length of the shortest path between them, say, the minimal number of links crossed to travel from node *i* to node *j*. If there is a link *i* → *j*, then *d*_*ij*_ = 1. If there is no other choice than going through an intermediate node *k* such that *i* → *k* → *j*, then *d*_*ij*_ = 2, and so on. When there exists no path connecting two nodes then *d*_*ij*_ = ∞. Often one finds groups of nodes in a network which are connected with each other (the graph distance between them is finite) but have no links to any other node outside the group. Such groups are referred to as connected components. The number and the size of the connected components indicate the degree of segregation of the network.

**Figure 7:**
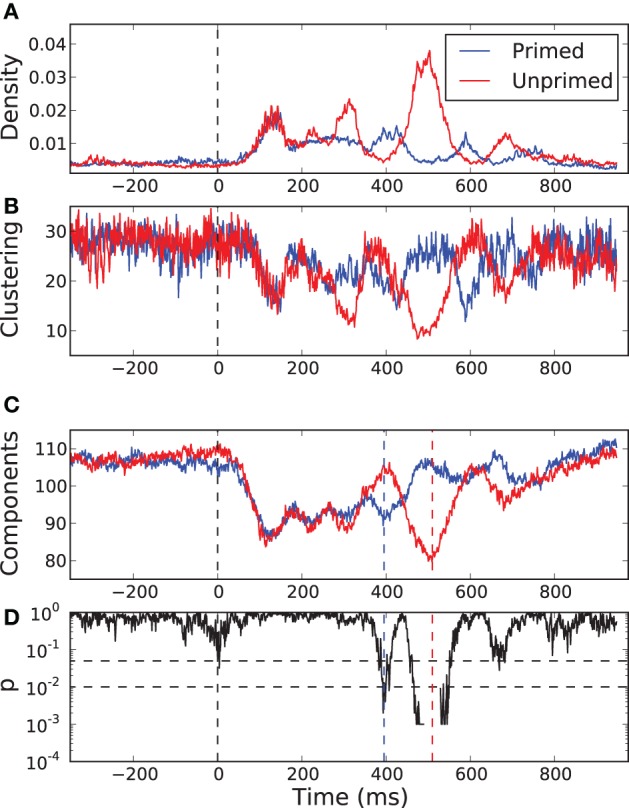
**Temporal evolution of some network properties in the primed condition (blue) and in the unprimed condition (red)**. **(A)** Density of links, **(B)** normalized clustering coefficient, and **(C)** the number of network components. All curves are the result of cross-subject averages. Zero time represents the moment of stimulus presentation, the second word. **(D)** *p*-value of a sliding permutation test of the cross-subject data of the number of components in **(C)**. It shows that the average curves for the primed and for the unprimed condition significantly deviate from each other at time intervals around 395 and 510 ms after stimulus presentation. The dash-dotted and dashed lines indicate the 0.05 and 0.01 level, respectively.

In Figures 7A,B we show the temporal evolution of the link density and the normalized clustering coefficient for the networks in both the primed (blue) and the unprimed (red) condition along the time course of the experimental measurements. Each curve is the average of the values across subjects. The zero time corresponds to the moment in which the second word was presented. Before the stimulus presentation all graph measures remain stable and the curves for both conditions take very similar values, as it is expected. The first 200 ms after word presentation correspond mainly to visual processing of the stimulus and both curves remain indistinguishable despite the variation in their shape. Between 300 and 700 ms after stimulus presentation the two curves undergo an epoch in which they largely deviate from each other, only to converge again at the end of the observation. This is precisely the time interval in which the cognitive processes happen.

We observe that the normalized clustering anti correlates with the density of links. Although the actual value of the clustering does increase with increasing link density (not shown), it becomes less significant than expected. In order to understand why this happens, we have to focus on the fragmentation of the network. As we referred previously, the identity condition in Equation (2) implies that the resulting networks are segregated into several network components. The clustering depends on the number and the sizes of those components.

In Figure 7C we show the temporal evolution of the number of components for both conditions. A sliding permutation test with 2000 permutations (Good, 2005) on the component number is included, Figure 7D. A permutation test is a non-parametric counterpart of a classical *t*-test that has fewer constraints on the distributions of the variables being tested. As it happens for the link density and for the clustering, in the pre-stimulus interval the curves follow each other and are statistically indistinguishable. The number of components is similar in both conditions and is almost as large as the number of electrodes, meaning that most of the nodes are functionally independent. With the beginning of the visual processing, shortly after stimulus onset, the number of components decays very fast and equally in both conditions. Both networks undergo a percolation process in which small components join together, indicating the onset of coordinated processing as a consequence of the visual input. The two curves start to diverge after 240 ms. The curve for the primed condition reaches a local minima at 395 ms followed by a rapid increase. This may be interpreted as the main cognitive processing finishing after 395 ms. The curve for the unprimed condition follows a similar pattern but delayed by 200 ms. Its minimum is more pronounced than the one of the primed condition, so more electrodes are involved in the coordinated processing. The delay is preceded by a short period (300–400 ms) in which the network of the unprimed condition is largely segregated.

**Figure 8:**
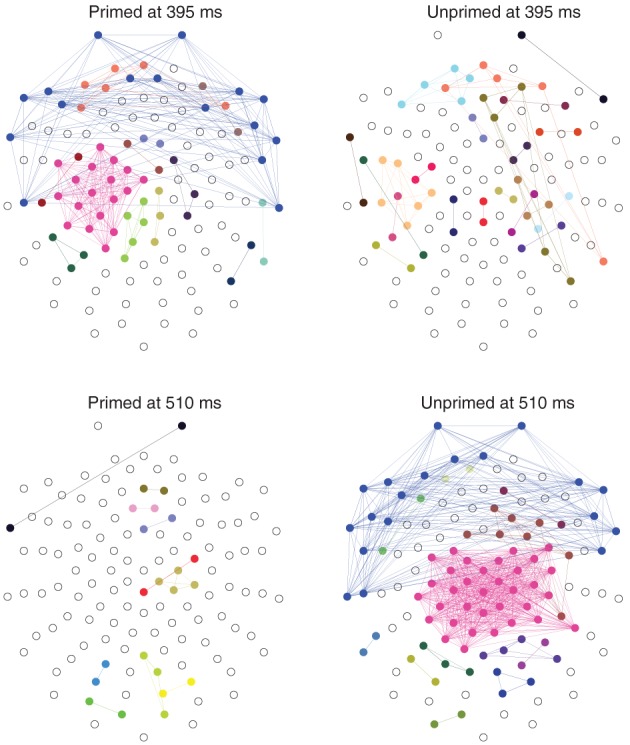
**Distribution of connected components on the scalp at the time points where the number of components maximally differs (395 and 510 ms post-stimulus)**. Electrodes belonging to the same component have the same color. Components of size 1 are left blank. The networks were computed from the grand average ERP using the same parameters as above ($\hat{d}$ = 8, τ = 15). The nose it toward the top.

The most significant differences between the two conditions happen at 395 ms and at 510 ms, with significance levels of *p* < 0.01. At those time points the topology of the networks are somewhat reversed, Figure 8. At 395 ms post-stimulus, the network in the primed condition is dominated by a slightly lateralized component, while the unprimed network is very segregated. At 510 ms post-stimulus, the network in the primed condition is almost fully segregated and the network of the unprimed condition is dominated by a large component, though it is more centralized than the dominant component in the primed condition at 395 ms.

In terms of ERP analysis the latency differences between the onset of peaks in the primed and in the unprimed conditions can be explained in two ways: on the one hand it could reflect the presence of two distinct processes that manifest at different time points. On the other hand, it is possible that we here observe the same cognitive process with a difference in latency. A point that would support the latter hypothesis to be true, is that the minimal number of components, 89 in the primed and 79 in the unprimed condition, does not differ significantly (*p* > 0.05). We are currently unable to decide which of these two explanations is more plausible, but future research will assess this difference in processing.

## 5. Conclusion

We have presented orpan, a method to estimate time-evolving functional connectivity from measured multivariate data. The method is based on the concept of order patterns, a symbolic representation of time series which is independent of the scale of the measurement and is almost unaffected by high-frequency fluctuations, e.g., additive noise. From a technical point of view, we can highlight several benefits of the method. First, the symbolic encoding is rather robust to non-stationarities and noise, and it is invariant with respect to a (slowly) increasing amplitude transformation—such as drifts in the data—which makes it very suitable for the analysis of electrophysiological data. Second, compared to other methods, the number of required parameters is very small, reducing the time required for searching in the parameter space. The only two parameters required are the embedding dimension *d*, which defines the length of the order patterns, and the embedding delay τ. Both parameters can be estimated using well-established algorithms. Finally, the approach is computationally very efficient and could be easily implemented to run in real time on dedicated systems or hardware, e.g., for brain-computer interfaces. The analytical complexity of the algorithm is $O(\frac{N^{2}}{2}T)$ where *N* in the number of nodes and *T* is the number of time points. The actual runtime of our (vectorized) implementation is linear with respect to *N* and *T*. For the electrophysiological data we used here (126 channels, 1400 time-points), it took approximately 30 seconds to estimate *d* and 1.4 seconds to estimate τ (per subject) on an off-the-shelf PC (code written in MATLAB). Once the parameters are fixed, it takes less than 2 seconds to compute all the 1295 functional networks for one subject in one condition.

We have discussed and illustrated by practical applications some of the issues that users might encounter when estimating the parameters *d* and τ. In an example of two coupled or uncoupled Lorenz attractors, our recommendation to use an embedding dimension of $\hat{d}$ = 2*d* + 2 proved useful to increase the accuracy of detecting functional links. The orpan method performs far better in discarding false positive functional connections than linear correlation.

We have illustrated the application of the method to analyze electrophysiological data using an example study of language processing. Here we restricted our analysis to the functional connectivity between the electrodes which is the simplest to obtain. Application to reconstructed brain-electric sources could help to better understand the interaction of cortical areas during cognitive processing.

In a previous study using the same electrophysiological data (Schinkel et al., 2011), we found that the formation of network components differed across conditions over a range of thresholds of weighted connectivity matrices. The previous approach had certain shortcomings: due to computational limitations we were tied to using fixed, pre-defined time windows for our analysis. Furthermore, the method used, joint recurrence plots, had a larger number of parameters, that are not easy to estimate (Schinkel et al., 2008). Now, the orpan method does not only reproduce our previous observations but it allows to overcome many of those limitations. It allows us to trace the temporal evolution of the connectivity revealing also a difference in latency between the two experimental conditions.

Future work will be dedicated to relax the identity condition in Equation (2) such that a sequence of weighted adjacency matrices can be obtained. This will require the use of symbolic similarity measures such as the Levenshtein distance (Levenshtein, 1966). While this may bring certain advantages, it is likely to require application of a threshold for network analysis. As the selection of a threshold implies some arbitrary choices, we refrain from this. At this point, we have shown the benefits of a straightforward and computationally efficient symbolic approach to estimate functional connectivity with temporal resolution.

### Conflict of interest statement

The authors declare that the research was conducted in the absence of any commercial or financial relationships that could be construed as a potential conflict of interest.

## Footnotes

^1^The actual dimension of a Lorenz system is *d* = 3, which is larger than the estimate. But when applying the method to experimental data we have to rely on estimates, which is why we used the estimated *d* = 2. For a reference we also calculated the results using *d* = 3, (see Table A1). The results do not change significantly.

## Appendix

### Comparison of the Lorenz system with *d* = 3

**Table A1:** **Summary of the detection rate analogous to Table 1 but using the analytical *d* = 3 and $\hat{d}$ = 8**.

**Case**	**Dimension**	**<Detection rate>**	
		**ORPAN**	**Correlation**
Coupled	*d* = 3	0.99	1.00
	$\hat{d}$ = 8	0.93	1.00
Uncoupled	*d* = 3	0.27^*^	0.75^*^
	$\hat{d}$ = 8	0.01^*^	0.44^*^

*Rates computed out of 1000 realizations. Asterisks denote the false positives in the uncoupled case*.

### Dependence on embedding dimension *d* and delay τ

We checked the dependence of results on the parameters *d* and τ. Summary plots are provided at http://people.physik.hu-berlin.de/~schinkel/supplements/frontiers.html
